# An overview of the Charcot foot pathophysiology

**DOI:** 10.3402/dfa.v4i0.21117

**Published:** 2013-08-02

**Authors:** Gökhan Kaynak, Olgar Birsel, Mehmet Fatih Güven, Tahir Öğüt

**Affiliations:** Cerrahpasa Faculty of Medicine, Department of Orthopedics and Traumatology, Istanbul University, Istanbul, Turkey

**Keywords:** Charcot foot, pathophysiology, diabetes mellitus, neuropathy, neuropeptides

## Abstract

Charcot arthropathy of the foot is a rare but devastating complication of diabetes that remains to be a challenging issue for the foot and ankle surgeons. Charcot foot fails to be an obvious diagnostic option that comes to mind, even in a pathognomonic clinical appearance. The rarity of the disorder, more common pathologies that mimic the condition, and the self-limiting prognosis deviate the clinician from the right diagnosis. The clinical challenges in the diagnosis of Charcot foot require in-depth investigations of its enigmatic nature to establish useful guidelines. Yet, this goal seems to be beyond reach, without a holistic view of the immense literature concerning the pathophysiology of the disorder. The primary objective of this article is to put together and review the recent advancements about the etiology and intrinsic mechanisms of diabetic Charcot foot.

Neuropathic arthropathy, also referred as Charcot arthropathy which was named after French neurologist Jean-Martin Charcot (1825–1893), is a progressive, denervation-induced degeneration of the foot and ankle joints (1). Considering devastating outcomes, such as eventual deformity which is almost always inevitable when untreated, the etiology and pathophysiology of this insidious disorder is vigorously studied in the literature as one of the core topics (2). A significant amount of content is amassing in the literature about this topic and this may be a sign of unclarity about the pathophysiology of Charcot arthropathy. Although numerous factors have been attributed as a contributor, the big picture is still not fully revealed.

A variety of causes are held responsible for triggering, amplifying, and converting the inflammatory processes which appears to be the major suspect at hand (3). Firm evidence was set forth that proinflammatory cytokines, especially the receptor activator of the nuclear factor-κB (RANK) ligand (RANKL) system is responsible for abnormally intense osteoclastogenesis (4). Increased osteoclastic activity is indicated for the excessive and unsupported bone turnover (5). The reciprocal relationship between the inflammation and repetitive traumas due to sensorial neuropathy has been substantially revealed. Yet, the rarity of the condition, its asymmetric involvement and its self-limiting progress still remain enigmatic (6). The purpose of this review is to put together asunder pieces of literature that aim to explain the condition from different perspectives and to develop a holistic approach to enlighten the nature of the inflammatory modulation.

Discovering the underlying disorder is a matter of utmost importance. When the diagnosis is Charcot arthropathy, in addition to meticulous treatment of the affected joint, the main goal should be the cure of the primary cause, if possible. Traumatic injuries (spinal cord injuries, peripheral nerve injuries), infections (syphilis, leprosy, yaws), disorders of neurological structures (myelomeningocele, syringomyelia, spina bifida), neurodegenerative diseases (amyloid neuropathy, neuropathies secondary to alcoholism and vitamin deficiency) or other neurological disorders such as congenital insensitivity to pain syndrome, steroid intake (post-renal transplant arthropathy, intra-articular steroid injections), and heavy-metal poisoning belong to the same cluster of diseases leading to destruction of afferent proprioceptive fibers (7–17). This is the core phenomenon that causes subsequent repetitive traumas to remain unrecognized. After numerous minor or major traumatic injuries, progressive wear results in microfractures, escalating into macrofractures that eventually end up in massive joint destruction with characteristic clinical presentation (Figs. 1 and 2) (17, 18).

**Fig. 1:**
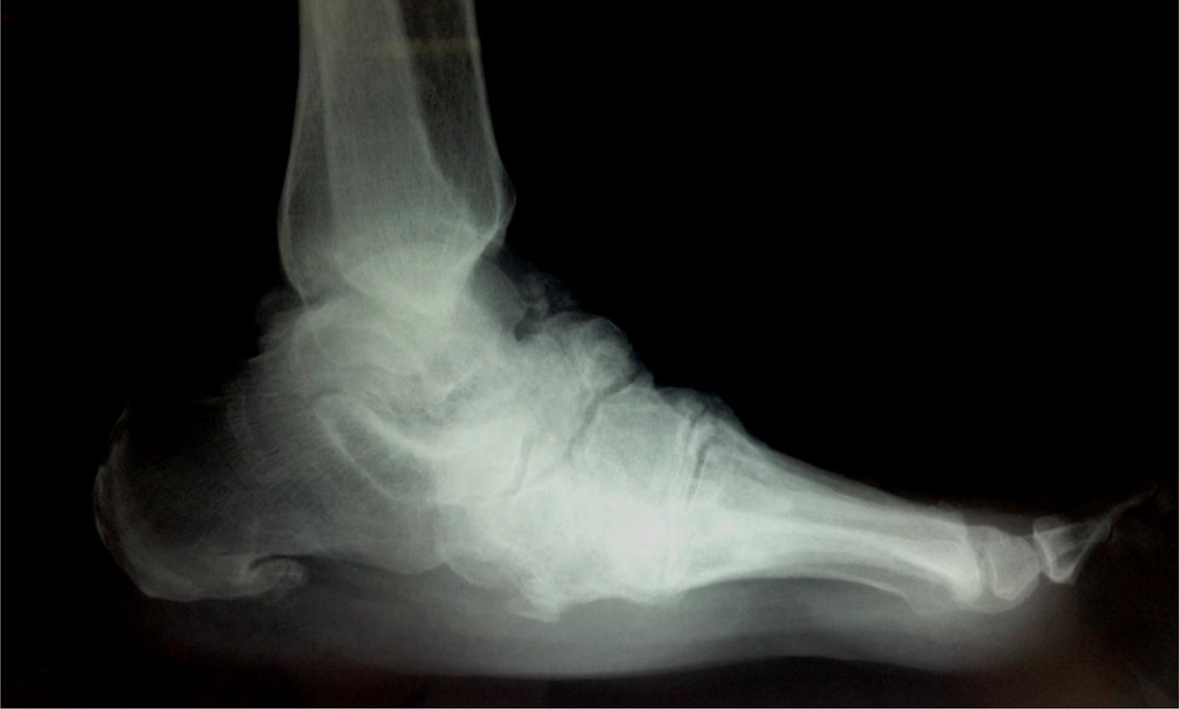
A 53-year-old diabetic female patient with a Charcot foot. (Source: Archives of Istanbul University, Cerrahpaşa Medical Faculty, Department of Orthopedics and Traumatology.)

**Fig. 2:**
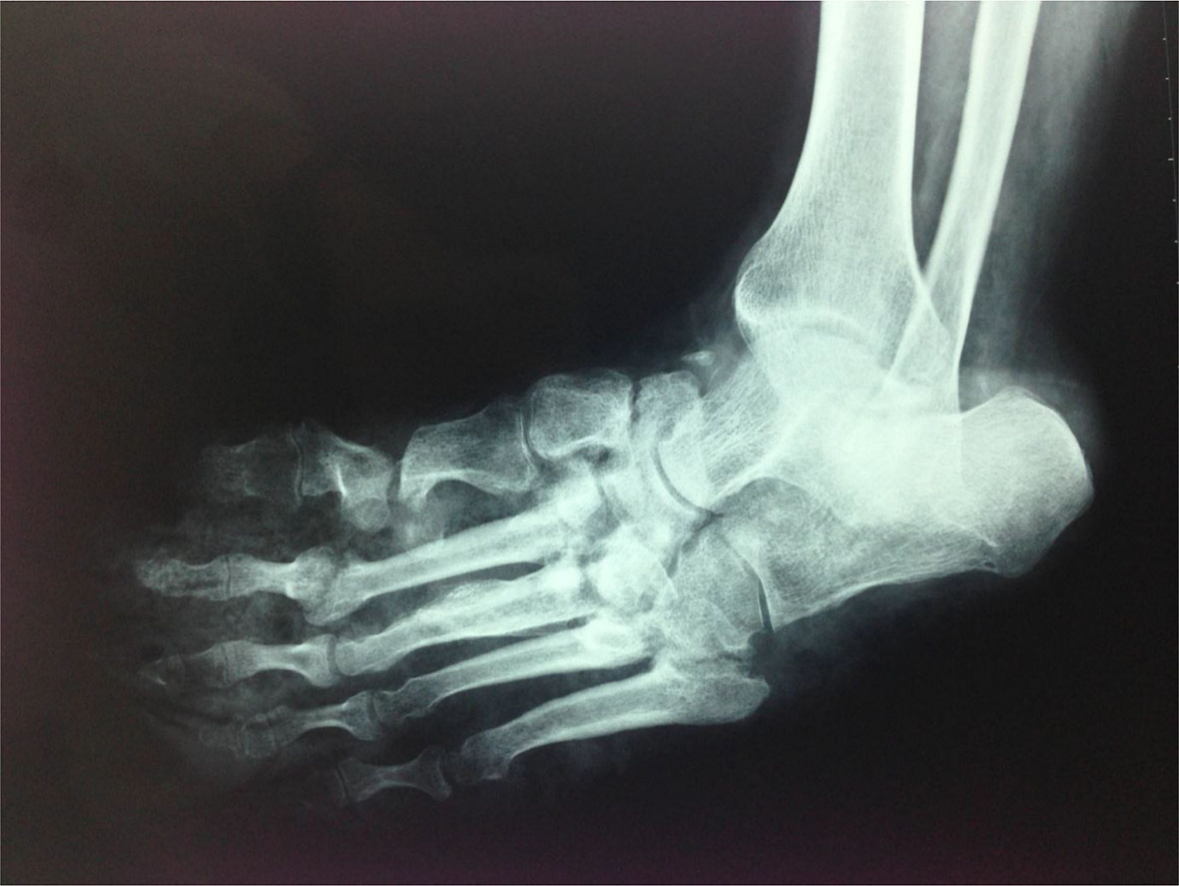
A 61-year-old diabetic male patient with a Charcot foot. (Source: Archives of Istanbul University, Cerrahpaşa Medical Faculty, Department of Orthopedics and Traumatology.)

However, recent studies demonstrated that the loss of proprioception is not the only etiological factor that leads to Charcot arthropathy (19). The intervention of certain external factors, usually unrecognized or irrelevant to the diabetes, or any other predisposing disease, is a factor that leads to a local increase in blood flow. Therefore, the patient's peripheral circulation should be capable of vasodilatating despite widespread atherosclerosis, which explains why Charcot foot is frequent in a relatively younger population. In summary, to develop a Charcot syndrome, it is understood that a patient should have a peripheral neuropathy to enhance a local inflammation that is triggered by a minor injury, infection, operation, or an earlier ulceration (1, 20).

Presently, diabetes is recognized as one of the most common cause of denervation-induced arthropathy worldwide (18, 20–23), the exception is for leprosy, which appears to be a more common cause of the Charcot foot in endemic areas (6, 7, 24). In the European Diabetes Centers study of complications in patients with insulin-dependent diabetes mellitus (EURODIAB IDDM), in which a considerable number of diabetic patients were included, the overall prevalence of neuropathy was 28% (25). Nevertheless, the incidence of Charcot arthropathy is relatively rare, even in neuropathic patients. Charcot arthropathy's estimated prevalence is approximately 1% of all neuropathic patients (20). Addressing this rarity, Jeffcoate explains that Charcot arthropathy develops or progresses in a small subset of patients where a number of predisposing factors coexist (26). The prevalence of diagnosed Charcot arthropathy in patients with diabetes is reported to be 0.08–7.5% (3). However, some studies suggest higher prevalence with as many as 13% of all diabetic patients and 29% of the neuropathic patients affected (22, 27). Due to the lack of specific markers or diagnostic criteria and Charcot artropathy's clinical resemblance to relatively more common disorders such as osteomyelitis, the diagnosis is missed or delayed in 25% of the cases (28). Yet, an increase in the incidence of Charcot arthropathy has been reported in recent studies. This increase can be explained by the progress made at the diagnostic modalities, the rising awareness of the condition amongst the physicians and caregivers, and the decreasing rate of lower limb amputations due to improved hospital cares (24).

A prospective study conducted in Singapore with 202 diabetic patients revealed that 42.1% of the patients had sensory neuropathy and 2% of them had Charcot arthropathy (29). The incidences of Charcot foot in type 1 and type 2 diabetes do not differ, although osteopenia, as a predisposing factor, appears to be more prevalent in type 1 (18, 20). However, Petrova et al. reported a difference in the presentation of Charcot arthropathy at type 1 and type 2 diabetic patients. In a more recent study, the same authors emphasized a relative preponderance of type 1 diabetes compared with type 2 (18, 30). Although the unilaterality of the condition is emphasized in many clinical studies, acute Charcot arthropathy is reported as bilateral in 9% of the patients (31). Moreover, after prospective computerized tomography examinations, bilateral neuroarthropathic changes are demonstrated in 75% of Charcot patients (17). Chisholm and colleagues suggest that obesity is also a predisposing factor for Charcot arthropathy since at least two thirds of Charcot patients are obese (22, 32).

Trauma is the most common etiological factor encountered in the pathogenesis of Charcot arthropathy and was reported to be present in 22–53% of the cases (6, 17, 18). Capillary leakage and subsequent formation of edema is the physiological response to blunt trauma (33). A higher energy trauma causes a disruption of marrow trabeculae leading to interstitial fluid and hemorrhage accumulation to marrow spaces, hence a bone bruise. When this condition occurs in the foot of a non-diabetic patient, it is painful and following the cessation of ambulation, local inflammation of the foot subsides. But in a neuropathic patient, the insensitive foot does not exhibit pain as appropriate. Thus, lack of required immobilization flares up the inflammatory cycle (26).

Osteomyelitis and surgery may initiate Charcot arthropathy in predisposing backgrounds (34). As mentioned before, osteomyelitis can imitate the clinical appearance of Charcot arthropathy and consequently lead to misdiagnosis and under-treatment. One third of the diabetic foot infections are complicated by osteomyelitis which is due to direct contamination from a soft, tissue ulcer (35). Osteomyelitis manifests with local inflammation and progressive destruction of the bone which in turn intensify the proinflammatory cascade.

Local surgery of the foot is suggested as one of the triggering factors of Charcot arthropathy (26, 36). Armstrong et al. retrospectively observed data from 55 acute Charcot arthropathy patients and reported that 4% of the patients had recent foot surgery as the only etiological factor (31). Charcot arthropathy may also follow injudicious immobilization after surgery, a long period of bed rest or casting (18). Disobedience to a forbidden weight bearing after foot surgery is also underlined in a case report as a possible cause of Charcot arthropathy (36).

Charcot arthropathy has also been reported following a simultaneous pancreas-kidney transplantation (SPKT) (37). In a more recent study where data from 130 SPKT patients without any previous history of Charcot arthropathy were analyzed retrospectively, six patients (4.6%) were diagnosed *de novo* Charcot arthropathy during the first year of transplantation where high glucocorticoid intake was the main factor leading to bone resorption and myofibril proteolysis (37).

## Pathophysiology

The well-respected neurotraumatic and neurotrophic theories have been relied on for a long time in order to clarify the pathogenesis of Charcot arthropathy. But none of these theories can explain this pathogenesis by themselves. Nevertheless, as the research advanced, neither the neurotrophic theory which was represented by Charcot himself (22) nor the neurotraumatic theory which was supported mainly by German scientists, have lost value. On the contrary, as complex biochemical processes revealed, the pathways of the two theories became more and more intertwined (22, 27).

Charcot, who first studied disturbed physiological processes that end up to neuropathic arthropathy, presented the neurovascular theory (38). He believed that irritation of the ‘trophic’ or vasomotor nerve centers caused an alteration of bone and joint nutrition. This theory has been carried further by his supporters, and now it suggests that autonomic neuropathy disregulates smooth muscle tonus on the arterial wall. Thus, it leads to a failure in vasoregulation and an increase in blood flow to the bone. Monocytes and osteoclasts storm the affected site and this accelerates the bone resorption rate resulting in osteopenia. Lower structural resistance cause minor traumas to end up in fractures, dislocations, and joint collapses (22). Volkman and Virchow confronted this theory, suggesting an insensate foot is prone to repetitive unrecognized traumas (38). In addition to this, loss of proprioception disables the protective factors and causes an abnormal joint loading. Multiple traumas and instability result in joint deterioration. The contribution of each of the theories to the pathogenesis of Charcot foot is broadly detailed and discussed below.

### Inflammation

Local inflammation is the indispensable factor that triggers the course of events on a predisposing environment. The physiological balance between the pro and anti-inflammatory cytokines that restrains the inflammatory response to a necessary extent is compromised in Charcot patients. Baumhauer's findings support this phenomenon based on the fact that in a Charcot patient the modulation of immune system is disturbed in countenance of proinflammatory cytokines (6). Whatever is the stimulant, the bone and soft tissues respond with an acute-phase release of proinflammatory cytokines, tumor necrosis factor-α (TNFα), and interleukin-1β (IL-1β). They have found an increase in the amount of TNF-α, IL-1β, and interleukin-6 (IL-6), whereas a decrease in the levels of interleukin-4 and interleukin-10 known as anti-inflammatory cytokines (39). An abnormally intense and prolonged inflammatory response is inevitable under these circumstances.

Increased amounts of proinflammatory cytokines, especially TNFα is found to be responsible for triggering another cytokine pathway that is centered on the polypeptide, the receptor activator of nuclear factor-κB (NF-κB) ligand (RANKL). As a member of the TNF superfamily, RANKL is the ligand that activates the receptor of NF-κB (RANK). The activation of RANK stimulates the intracellular pathways that end up by formation of nuclear transcription factor NF-κB. The expression of NF-κB induces osteoclast precursor cells to differentiate into mature osteoclasts (20, 21). Thus, NF-κB pathway is implicated in the excessive osteoclastic activity in diabetic Charcot arthropathy (40, 41) along with its involvement in many conditions that manifest with osteolysis including glucocorticoid-induced osteoporosis, metastatic malignancy, periodontitis, prosthesis-related osteolysis, and rheumatoid arthritis (20, 26).

**Fig. 3:**
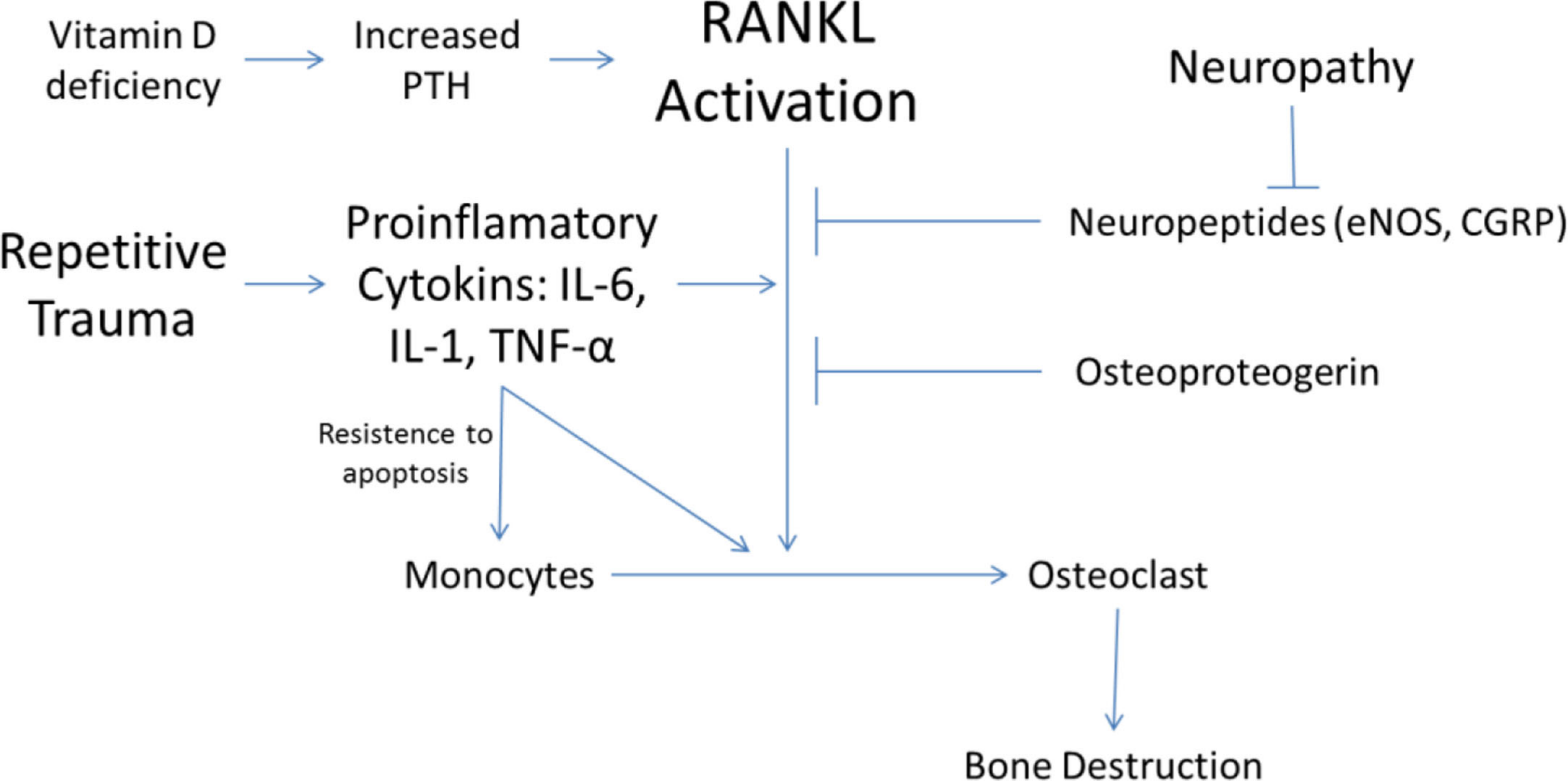
RANKL pathway in the pathophysiology of Charcot arthropathy. (The copyright of the figure belongs to the authors.)

RANKL activity is antagonized by Osteoprotegerin (OPG), a soluble glycoprotein decoy receptor for RANK ligand which effectively neutralizes its effects (20). OPG's expression is induced by NF-κB, as a self-limiting agent of its proinflammatory function. The patients with Charcot arthropathy displayed elevated RANKL/OPG ratios fuelling the progression of the inflammation (Fig. 3) (41, 42).

Osteoclasts play a key role in the course of Charcot arthropathy as executer cells, responsible for imbalanced bone turnover and eventually osteolysis. Macrophage colony stimulating factor (M-CSF) is required for precursor cells in myeloid lineage to differentiate into bone marrow macrophages, but the activation of NF-κB is crucial for their further differentiation, fusion into multinucleated osteoclasts, activation, and survival (41). However, the first cell line to dysfunction seems to be the monocytes, the precursor cells of osteoclasts. With high levels of proinflammatory cytokines, monocytes stimulate T lymphocytes in an exaggerated way. In addition to this, monocytes obtained from Charcot patients present reduced secretion of anti-inflammatory cytokines, and increased resistance to apoptosis (21, 42). This resistance provided mainly by IL-1β and TNFα causes the persistence of the abnormally intense and prolonged inflammatory response (3). Ndip et al. reported that IL-8 and Granulocyte-Colony Stimulating Factor (GCSF) were inducing monocytes into an osteoclastic differentiation along with the RANKL/RANK pathway (41).

Pitocco et al. encountered a significant decrease in the circulating levels of IGF-1 while investigating biphosphonate's efficacy in Charcot patients (43). IGF-1 is a mediator of vasodilatation, and biphosphonate's reducing effect over IGF-1 could have a beneficial contribution to restrain proceeding inflammation.

### Neuropeptides

It has long been suspected that the central nervous system intervenes with the regulation and/or the modulation of the bone metabolism (44). This function is mediated through peptides that are synthesized in unmyelinated sensory neurons and secreted from their peripheral terminals in the bone tissue. However, this function differs from the synaptic transmission as the signal transduction by the neuropeptides is thought to be non-synaptic, diffuse, and slow (45). The essential role of neuropeptides in the bone metabolism is thoroughly studied by Offley et al. (46). They reported that capcaisin-induced depletion of neuropeptides such as Substance P (SP) and Calcitonin Gene-Related Peptide (CGRP) in unmyelinated sensory neurons of adult rats resulted in an increased bone loss and fragility. The authors suggested that this effect could be reversed by daily injections of CGRP (46).

CGRP is the most studied neuropeptide since evidences suggest a direct action of CGRP regulating the cellular activities of osteoblasts. CGRP is shown to bind to its own receptor and to increase intracellular cyclic AMP and calcium in osteoblastic cells. Moreover, CGRP stimulates cell proliferation, synthesis of cytokines, synthesis of growth factors, and synthesis of collagen (40). Most of the CGRP present in the circulation is released via non-synaptic secretion from small sensory nerve terminals of unmyelinated C type and small myelinated A-δ-type nerve fibers. These fibers are abundantly located in the periosteum, bone tissue, and bone marrow especially in the epiphyseal trabecular bone (45). CGRP is also shown to be expressed by osteoblasts endogenously, suggesting the existence of an autocrine loop (45). CGRP has been shown to inhibit proinflammatory cytokine production and increase the release of IL-10 by monocytes. Denervated Charcot foot is deprived of CGRP release thus an important source of anti-inflammatory impulse is compromised (21, 47).

Nitric oxide (NO) is a free radical gas that functions as a secondary messenger molecule in many biological pathways. Several studies suggest that NO has a reciprocal effect on the modulation of bone metabolism (48). NO's inhibitory effect over the osteoclasts is demonstrated in animal studies, observing NO being able to induce apoptosis of pre-osteoclasts and decrease the resorbtive action of the mature osteoclasts in mice (49). Diminished expression of eNOS is observed in Charcot patients, which leads to a suppression of the osteoclast activity and contributes to a marked increase in the fragility of osteoporotic bone (48). More recently Tan et al. studied the biomechanical effects of the pulsatile fluid flow (PFF) through the periosteocytic canaliculi (50). They demonstrated that PFF plays a major role in preventing osteocyte apoptosis, and this effect is mediated by NO. Osteoclasts are attracted by apoptotic osteocytes resulting in bone resorption. Thus, insufficient amounts of NO production might induce osteocytes to apoptosis, enhancing indirectly the osteoclast function. Other studies supported NO's biphasic effect on osteoclasts, suggesting that low concentrations of NO potentiate bone resorption while higher concentrations are inhibitory (51).

Sympathetic innervation of the bone and bone marrow is demonstrated by monitorization of thyrosine hydroxylase activity, the rate-limiting enzyme of catecholamine synthesis (46). Noradrenergic innervation of the bone tissue is effective not only in regulating blood flow but also in the modulation of osteoblastic and osteoclastic cell metabolism. Osteoblasts express β-2 adrenergic receptors. Moreover, noradrenalin increases alkaline phosphatase activity and proliferation through α-1 receptors expressed on the osteoblasts (23).

### Microvascular structure and bone turnover

When Charcot first described the neuropathic arthropaty, he implicated the increase in bone perfusion secondary to the sympathetic denervation as responsible for bone resorption (38). Jeffcoate improves this hypothesis further by suggesting that Charcot arthropathy requires the coexistence of a dense neuropathy with a relatively intact peripheral circulation (26). The rarity of Charcot arthropathy may be explained by the fact that it affects only the limbs with the capacity to mount an inflammatory response, in other words, to retain the ability to increase blood flow in response to a particular stimulus (20). Baker et al. studied the rate of maximum microvascular hyperemia (MMH) in patients with diabetic neuropathy and diabetic Charcot arthropathy and found that in Charcot patients, MMH is relatively preserved and significantly higher than patients with neuropathy alone (52). Similarly, Shapiro et al. demonstrated increased skin blood flow and vasomotion in both healthy control and Charcot subjects, in contrast with diabetic neuropathy patients (53). Those findings suggest that Charcot patients preserve the ability to vasodilate as opposed to patients with diabetic neuropathy alone, and it may be an explanation why all patients with diabetic neuropathy do not develop Charcot. However, peripheral arterial disease seems to have a protective effect on the development of Charcot arthropathy (24). This is probably due to limited vasodilation capacity of the affected arteries.

Sympathetic vascular denervation increases local arterio-venous shunt flow, which functions in body thermoregulation in physiological conditions. Unregulated shunts increase venous pressure and enhance fluid filtration through capillary leakage (24). Consequently, deep tissue edema increases intra-compartmental pressure, compromises microcirculation, and causes a deep tissue ischemia (54). Moreover, extensive connective tissue edema impairs tensile strength and stability of tendons and ligaments, predisposing the joints to subluxations and dislocations.

**Fig. 4:**
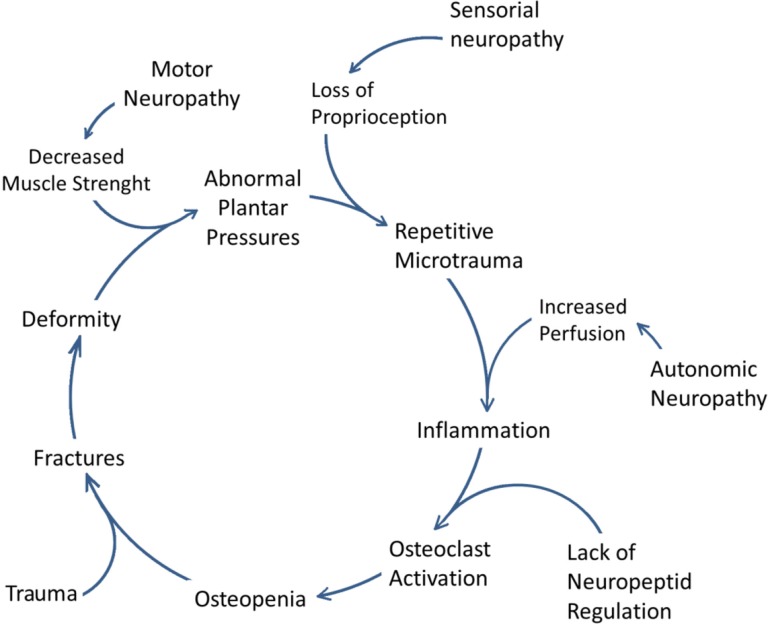
Cycle of pathophysiology of Charcot osteoarthropathy. (The copyright of the figure belongs to the authors.)

In Charcot arthropathy patients, exaggerated osteoclast activity leads to an imbalance of constant remodeling processes. An increase in alkaline phosphatase and collagen residues, indicative of osteoclast activity, were observed in the patients with acute Charcot arthropathy (55, 56). However, in a recent study La Fontaine suggested the possibility of a preexisting abnormal bone structure predisposing diabetic patients for Charcot foot (5). They have conducted histological examination of bone specimens obtained from diabetic patients and observed a distorted microstructure with fewer trabeculae and fewer cells. Thus, the authors argued that the degenerative changes in the bone micro-architecture may not be a consequence, but a cause of Charcot arthropathy (Fig. 4) (5).

Although a low bone mineral density (BMD) is observed in patients with type 1 diabetes, the BMD is similar to or higher in type 2 diabetic patients than in non-diabetic subjects (5). Yet, Charcot foot's prevalence does not seem to preponderate between types of diabetes (18, 20). Christensen et al. reported significantly lower BMD values obtained from the affected foot of chronic Charcot patients whereas no difference was in the calcaneal BMD between acute Charcot patients and the control group. Therefore, Charcot arthropathy does not seem to affect the skeleton in general (57). Moreover, biochemical indicators of bone turnover were also evaluated in the same study and statistically significant differences in osteocalcin concentrations reflecting increased bone turnover were demonstrated in acute Charcot foot (56).

A recent study demonstrated the development of Charcot arthropathy after administration of high doses of glucocorticoids (37). Glucocorticoids affect the bone turnover in countenance of resorption, and decreased bone formation may trigger or worsen Charcot arthropathy.

Vitamin D deficiency is accused of being preliminary to the development of Charcot arthropathy (37). Hypocalcaemia occurs in vitamin D deficiency and this stimulates parathyroid hormone (PTH) which in turn depletes calcium from the bone causing osteopenia. The level of 1,25(OH)_2_D_3_ is significantly lower in diabetic patients and this decrease results in a less mineralized bone formation, a smaller growth plate and an inadequate turnover, all being reversed by insulin treatment (49).

### Hyperglycemia

The expression of RANKL is proven to be closely associated with metabolic consequences of the diabetes. Increased blood sugar potentiates free radical formation, hyperlipidemia and advanced glycation end-products (AGEs), triggering the RANK/RANKL cytokine system (6). Moreover, an *in vitro* inhibitory effect of physiological concentrations of insulin on the NF-κB and monocyte chemoattractant protein 1 (MCP-1) is also reported (57). This non-RANKL inhibitory mechanism is impaired in diabetic patients who are deprived of insulin. However, those pathways may not be majorly effective since the Charcot arthropathy is rare even in diabetic neuropathy patients (58).

Hyperglycemia denaturates tendons and ligaments through a non-enzymatic collagen glycation. This phenomenon could cause tendon shortenings and thus, redistribution of the plantar pressures abnormally (17). Moreover, since collagen is a structural component of the bone, AGE-related modifications of collagen, may impair the mechanical properties of bone itself, predisposing it to fractures and dislocations (49, 59) (Figs. 3 and 4).

### Genetics

Genes regulating OPG/RANK/RANKL axis and their polymorphisms have already been indicated in the pathogenesis of the osteoporosis (60). A correlation between diabetic Charcot arthropathy and OPG gene polymorphisms is first suggested by Pitocco et al. (61). They have investigated single-nucleotide polymorphisms (SNPs) on the OPG gene and found two variations that could result in both qualitative and quantitative alterations of OPG. A strong association with Charcot arthropathy and the polymorphisms of those alleles were also demonstrated. Recently, Korzon-Burakowska et al. supported this association in their study conducted in the Polish population (28). Their results agreed with the previous study conducted in Italy (61), despite the differences in alleles between Charcot patients and control groups did not match among the two populations.

## Discussion

The pathophysiological process that leads to an acute Charcot foot episode is a very dynamic research topic. New studies are added to the immense pool of literature on this topic aiming to clarify and prevent the onset and progression of this devastating complication. Recent advances on the subject demonstrated that the neurotraumatic and neurotrophic theories both play a part and contribute to development of the Charcot foot. However, the question that still remains is why all patients with diabetic neuropathy do not develop a Charcot foot? A reasonable explanation to this question may enlighten the pathogenesis substantially and thus, set forth a possible way to avoid its onset.

In recent studies, many authors have emphasized the disturbance of the inflammatory cycle in the core of Charcot foot pathophysiology. Usually minor injuries which are even unrecognized, local infection or a minor surgery may prompt this sequence of events. Without the protective behavior ensured by the pain, in the insensate, neuropathic foot of a diabetic patient this cycle is flared up by repeated traumatic events. Genetic variations that affect the balance between pre- and anti-inflammatory chemo-attractants may predispose a patient to Charcot foot (28, 61). This theory which has been proposed by two different studies conducted in different populations is a breakthrough for researchers who dedicated their work to Charcot foot. Genetic variations can explain a patient's tendency to pro-inflammatory status, and ultimately may put forth a solid answer why a majority of neuropathic patients are spared from Charcot arthropathy.

Finally, it is often difficult to isolate and experiment the contribution of a single factor in this complex and multifactorial phenomenon. This fact precludes the construction of experimental models to reveal single factor contributions or gathering of adequate (appropriate) control groups for blinded control studies. This suggestion can be adapted to Charcot foot that develops as a result of a subsequent dysfunction concerning different systems which cannot be separated and observed solely. This stalemate can be addressed with prospective studies and a larger number of patients.

## References

[1] Jeffcoate W (2008). The causes of the Charcot syndrome. Clin Podiatr Med Surg.

[2] van der ven A, Chapman CB, Bowker JH (2009). Charcot neuroarthropathy of the foot and ankle. J Am Acad Orthop Surg.

[3] Larson SA, Burns PR (2012). The pathogenesis of Charcot neuroarthropathy: current concepts. Diabet Foot Ankle.

[4] Mabilleau G, Petrova N, Edmonds M, Sabokbar A (2008). Increased osteoclastic activity in acute Charcot's osteoarthropathy: the role of receptor activator of nuclear factor-kappa B ligand. Diabetologia.

[5] La Fontaine J, Shibuya N, Sampson HW, Valderrama P (2011). Trabecular quality and cellular characteristics of normal, diabetic, and Charcot bone. J Foot Ankle Surg.

[6] Jeffcoate WJ, Game F, Cavanagh PR (2005). The role of proinflammatory cytokines in the cause of neuropathic osteoarthropathy (acute Charcot foot) in diabetes. Lancet.

[7] Rostom S, Bahiri R, Mahfoud-Filali S, Hajjaj-Hassouni N (2007). Neurogenic osteoarthropathy in leprosy. Clin Rheumatol.

[8] Panagariya A, Sharma AK (2012). Bilateral Charcot arthropathy of shoulder secondary to syringomyelia: an unusual case report. Ann Indian Acad Neurol.

[9] Hatzis N, Kaar K, Wirth MA, Toro F, Rockwood CA (1998). Neuropathic arthropathy of the shoulder. J Bone Joint Surg Am.

[10] Kenan S, Lewis MM, Main WK, Hermann G, Abdelwahab IF (1993). Neuropathic arthropathy of the shoulder mimicking soft tissue sarcoma. Orthopedics.

[11] Atalar AC, Sungur M, Demirhan M, Ozger H (2010). Neuropathic arthropathy of the shoulder associated with syringomyelia: a report of six cases. Acta Orthop Traumatol Turc.

[12] Paliwal VK, Singh P, Rahi SK, Agarwal V, Gupta RK (2012). Charcot knee secondary to lumbar spinal cord syringomyelia: complication of spinal anesthesia. J Clin Rheumatol.

[13] Chopra K, Tiwari V (2012). Alcoholic neuropathy: possible mechanisms and future treatment possibilities. Br J Clin Pharmacol.

[14] Altenburg N, Joraschky P, Barthel A, Bittnert A (2011). Alcohol consumption and other psycho-social conditions as important factors in the development of diabetic foot ulcers. Diabet Med.

[15] Koike H, Sobue G (2006). Alcoholic neuropathy. Curr Opin Neurol.

[16] Zambelis T, Karandreas N, Tzavellas E (2005). Large and small fibre neuropathy in chronic alcohol-dependent subjects. J Peripher Nerv Syst.

[17] Gouveri E, Papanas N (2011). Charcot osteoarthropathy in diabetes: a brief review with an emphasis on clinical practice. World J Diabet.

[18] Petrova NL, Edmonds ME (2008). Charcot neuro-osteoarthropathy – current standards. Diabetes Metab Res Rev.

[19] Chantelau E, Onvlee GJ (2006). Charcot foot in diabetes: farewell to the neurotrophic theory. Horm Metab Res.

[20] Jeffcoate W (2008). Charcot neuro-osteoarthropathy. Diabetes Metab Res Rev.

[21] Uccioli L, Sinistro A, Almerighi C, Ciaprini C (2010). Proinflammatory modulation of the surface and cytokine phenotype of monocytes in patients with acute Charcot foot. Diabetes Care.

[22] Chisholm KA, Gilchrist JM (2011). The Charcot joint: a modern neurologic perspective. J Clin Neuromusc Dis.

[23] Jones KB, Mollano AV, Morcuende JA (2004). Bone and brain: a review of neural, hormonal, and musculoskeletal connections. Iowa Orthop J.

[24] Rajbhandari SM, Jenkins RC, Davies C (2002). Charcot neuroarthropathy in diabetes mellitus. Diabetologia.

[25] Tesfaye S, Stevens LK, Stephenson JM, Fuller JH (1996). Prevalence of diabetic peripheral neuropathy and its relation to glycaemic control and potential risk factors: the EURODIAB IDDM complications study. Diabetologia.

[26] Jeffcoate WJ (2005). Abnormalities of vasomotor regulation in the pathogenesis of the acute Charcot foot of diabetes mellitus. Int J Low Extrem Wounds.

[27] Slater RA, Ramot Y, Buchs A (2004). The diabetic Charcot foot. Isr Med Assoc J.

[28] Korzon–Burakowska A, Jakóbkiewicz-Banecka J, Fiedosiuk A, Petrova N (2012). Osteoprotegerin gene polymorphism in diabetic Charcot neuroarthropathy. Diabet Med.

[29] Nather A, Bee CS, Huak CY, Chew JLL (2008). Epidemiology of diabetic foot problems and predictive factors for limb loss. J Diabet Complications.

[30] Petrova NL, Foster AV, Edmonds ME (2004). Difference in presentation of Charcot osteoarthropathy in type 1 compared with type 2 diabetes. Diabetes Care.

[31] Armstrong DG, Todd WF, Lavery LA, Harkless LB, Bushman TR (1997). The natural history of acute Charcot's arthropathy in a diabetic foot specialty clinic. J Am Pediatr Med Assoc.

[32] Pinzur MS (1999). Benchmark analysis of diabetic patients with neuropathic (Charcot) foot deformity. Foot Ankle Int.

[33] Chantelau E, Richter A, Schmidt-Grigoriadis P, Scherbaum WA (2006). The diabetic Charcot foot: MRI discloses bone stres injury as trigger mechanism of neuroarthropathy. Exp Clin Endocrinol Diabetes.

[34] Ndip A, Jude EB, Whitehouse R, Prescott M, Boulton AJ (2008). Charcot neuroarthrtopathy triggered by osteomyelitis and/or surgery. Diabet Med.

[35] Giurato L, Uccioli L (2006). The diabetic foot: Charcot joint and osteomyelitis. Nucl Med Commun.

[36] Aragón-Sánchez J, Lázaro-Martínez JL, Hernández-Herrero MJ (2010). Triggering mechanisms of neuroarthropathy following conservative surgery for osteomyelitis. Diabet Med.

[37] Rangel ÉB, Sá JR, Gomes SA, Carvalho AB, Melaragno CS (2012). Charcot neuroarthropathy after simultaneous pancreas–kidney transplant. Transplantation.

[38] Charcot JM (1868). Sur quelques arthropathies qui paraissent dependre d'une lésion du cerveau ou de la mouelle épinière. [On some arthropathies apparently related to a lesion of the brain or spinal cord]. Arch Physiol Norm Pathol.

[39] Baumhauer JF, O'Keefe RJ, Schon LC, Pinzur MS (2006). Cytokine induced osteoclastic bone resorption in Charcot arthropathy: an immunohistochemical study. Foot Ankle Int.

[40] Wang L, Shi X, Zhao R, Halloran BP, Clark DJ (2010). Calcitonin-gene-related peptide stimulates stromal cell osteogenic differentiation and inhibits RANKL induced NF-kappaB activation, osteoclastogenesis and bone resorption. Bone.

[41] Ndip A, Williams A, Jude EB, Serracino-Inglott F (2011). The RANKL/RANK/OPG signaling pathway mediates medial arterial calcification in diabetic charcot neuroarthropathy. Diabetes.

[42] Mangan DF, Welch GR, Wahl SM (1991). Lipopolysaccharide, tumor necrosis factor-alpha, and IL-1 beta prevent programmed cell death (apoptosis) in human peripheral blood monocytes. J Immunol.

[43] Pitocco D, Ruotolo V, Caputo S, Mancini L, Collina CM (2005). Six-month treatment with alendronate in acute Charcot neuroarthropathy: a randomized controlled trial. Diabetes Care.

[44] Irie K, Hara-Irie F, Ozawa H, Yajima T (2002). Calcitonin gene-related peptide (CGRP) -containing nerve fibers in bone tissue and their involvement in bone remodeling. Microsc Res Tech.

[45] Imai S, Matsusue Y (2002). Neuronal regulation of bone metabolism and anabolism: calcitonin gene-related peptide-, substance P-, and tyrosine hydroxylase-containing nerves and the bone. Microsc Res Tech.

[46] Offley SC, Guo TZ, Wei T, Clark JD, Vogel H, Lindsey DP (2005). Capsaicin-sensitive sensory neurons contribute to the maintenance of trabecular bone integrity. J Bone Miner.

[47] Akopian A, Demulder A, Ouriaghli F, Corazza F, Fondu P, Bergmann P (2000). Effects of CGRP on human osteoclast-like cell formation: a possible connection with the bone loss in neurological disorders?. Peptides.

[48] La Fontaine J, Harkless LB, Sylvia VL, Carnes D, Heim-Hall J, Jude E (2008). Levels of endothelial nitric oxide synthase and calcitonin gene-related peptide in the Charcot foot: a pilot study. J Foot Ankle Surg.

[49] Blakytny R, Spraul M, Jude EB (2011). Review: the diabetic bone: a cellular and molecular perspective. Int J Low Extrem Wounds.

[50] Tan SD, Bakker AD, Semeins CM, Kuijpers-Jagtman AM, Klein-Nulend J (2008). Inhibition of osteocyte apoptosis by fluid flow is mediated by nitric oxide. Biochem Biophys Res Commun.

[51] Nilforoushan D, Gramoun A, Glogauer M, Manolson MF (2009). Nitric oxide enhances osteoclastogenesis possibly by mediating cell fusion. Nitric Oxide.

[52] Baker N, Green A, Krishnan S, Rayman G (2007). Microvascular and C-fiber function in diabetic Charcot neuroarthropathy and diabetic peripheral neuropathy. Diabetes Care.

[53] Shapiro SA, Stansberry KB, Hill MA, Meyer MD (1998). Normal blood flow response and vasomotion in the diabetic Charcot foot. J Diabetes Complications.

[54] Schaper NC, Huijberts M, Pickwell K (2008). Neurovascular control and neurogenic inflammation in diabetes. Diabetes Metab Res Rev.

[55] Childs M, Armstrong DG, Edelson GW (1998). Is Charcot arthropathy a late sequela of osteoporosis in patients with diabetes mellitus?. J Foot Ankle Surg.

[56] Christensen TM, Bülow J, Simonsen L, Holstein PE, Svendsen OL (2010). Bone mineral density in diabetes mellitus patients with and without a Charcot foot. Clin Physiol Funct Imaging.

[57] Aljada A, Ghanim H, Saadeh R, Dandona P (2001). Insulin inhibits NF-κB and MCP-1 expression in human aortic endothelial cells. J Clin Endocrinol Metab.

[58] Rogers LC, Frykberg RG, Armstrong DG, Boulton AJM, Edmonds M (2011). The Charcot foot in diabetes. Diabetes Care.

[59] Viguet-Carrin S, Garnero P, Delmas PD (2006). The role of collagen in bone strength. Osteoporos Int.

[60] Kim JG, Kim JH, Kim JY, Ku SY, Jee BC (2007). Association between osteoprotegerin (OPG), receptor activator of nuclear factor-kappaB (RANK), and RANK ligand (RANKL) gene polymorphisms and circulating OPG, soluble RANKL levels, and bone mineral density in Korean postmenopausal women. Menopause.

[61] Pitocco D, Zelano G, Gioffrè G, Di Stasio E, Zaccardi F (2009). Association between osteoprotegerin G1181C and T245G polymorphisms and diabetic Charcot neuroarthropathy: a case-control study. Diabetes Care.

